# Nanostructured Bismuth Film Electrode for Detection of Progesterone

**DOI:** 10.3390/s18124233

**Published:** 2018-12-02

**Authors:** Tanja Zidarič, Vasko Jovanovski, Samo B. Hočevar

**Affiliations:** Department of Analytical Chemistry, National Institute of Chemistry, Hajdrihova 19, SI-1000 Ljubljana, Slovenia; tzidaric@gmail.com

**Keywords:** nanostructured bismuth film electrode, nsBiFE, adsorptive cathodic stripping voltammetry, progesterone, premature birth

## Abstract

Progesterone is an important hormone responsible, among others, for maintaining pregnancy via inhibition of uterus muscles activity; thus, following its concentration levels in pregnant women is of immense importance in the endeavor to prevent premature birth. In this work, the nanostructured bismuth film electrode (nsBiFE) was studied for detection of progesterone in neutral medium. Due to the ability to accumulate progesterone at the nsBiFE, the adsorptive cathodic stripping voltammetry was beneficially exploited. The nsBiFE was prepared on the surface of a glassy carbon supporting electrode and several parameters influencing the detection of progesterone were investigated. The nsBiFE exhibited superior electroanalytical characteristics in comparison to other bismuth-based electrodes and unmodified glassy carbon electrode together with satisfactory response toward low concentrations of progesterone, which are consistent with clinically significant levels.

## 1. Introduction

Progesterone is a natural steroid and an essential hormone for stabilization and maintaining pregnancy. In mammals it is formed from cholesterol, primarily in ovaries, placenta, and the adrenal glands where it is synthesized after ovulation [1,2,3,4,5]. In addition, progesterone is a precursor of other essential hormones (estrogen, testosterone) in the adrenal glands, influencing their activity [6]. Premature birth represents a challenge in the field of perinatology as it is the main cause of infant death or risk of serious medical conditions. The global statistics show that the number of premature births is between 5% and 10%. Currently, there is an increase in reported premature births; mainly due to older pregnant women, use of auxiliary reproductive techniques, increased bodyweight index and various infections [7,8]. Progesterone plays an important role in maintaining pregnancy via inhibition of uterus muscles activity, otherwise activated by oxytocin and prostaglandins that can trigger a premature birth [7,9,10]. Studies have shown that monitoring the levels of progesterone in pregnant women gives invaluable information for prognosis of normal embryo development in early pregnancy [9,11]. In the study by Fonaseca et al., it was suggested that low serum concentrations of progesterone are linked to a higher incidence of preterm births [12].

In clinical diagnostics, several detection methods were developed for detecting progesterone, including spectroscopic, spectrometric (hyphenated with chromatography), enzymatic immunoassay, etc [13,14,15,16]. More recently, several examples of electrochemical immunoassay emerged, together with a few cases of direct voltammetric detection performed on mercury, tin and various carbon electrodes in alkaline media [3,4,17,18,19,20].

Bismuth film electrode, that exhibits similar properties to its mercury analogue, was also employed in the electrochemical determination of progesterone (in pharmaceutical formulation) in alkaline medium, i.e., in a Britton-Robinson buffer solution of pH 12.0. The cyclic voltammetric study revealed two signals at ca. −1.5 V and −1.7 V, which were attributed to a quasi-reversible redox process of progesterone. An electrochemical adsorption-controlled reaction was suggested involving one electron transfer.

Herein, we demonstrated a preliminary study of a nanostructured bismuth film electrode (nsBiFE) for adsorptive cathodic stripping voltammetric detection of progesterone in a neutral medium exhibiting favorable electroanalytical characteristics, thus holding great promise considering its potential application for rapid monitoring of progesterone in pharmaceutical formulations and in clinical diagnostics.

## 2. Materials and Methods

Electrochemical measurements were performed in an electrochemical cell using an Autolab PGSTAT12 instrument (Eco Chemie, Utrecht, The Netherlands). A glassy carbon supporting electrode (modified with bismuth film) was used as the working electrode, an Ag/AgCl/KCl (satd.) was employed as the reference electrode, and a platinum wire as the counter electrode.

A standard solution of bismuth(III) (1000 mg L^−1^ in 2–3% HNO_3_) was obtained from Merck, and progesterone was provided by Sigma-Aldrich. Acetate buffer solution (0.1 M, pH 4.5) used for the preparation of nsBiFE, and sodium phosphate buffer solution (Na-PBS, 0.1 M, pH 7.1) which served as the supporting electrolyte, were prepared by mixing adequate amounts of acetic acid and sodium acetate trihydrate (for acetate buffer solution), and sodium hydrogen phosphate and sodium dihydrogen phosphate (for Na-PBS). Water was purified via Elix 10/Milli-Q Gradient unit (Milipore, Bedford, MA, USA).

(i) The in-situ preparation of BiFE was carried out via the addition of 1 mg L^−1^ Bi(III) directly into the measurement solution together with the analyte. During in-situ preparation of BiFE, the bismuth film was deposited on the electrode surface simultaneously with the analyte using accumulation potential of −1.0 V. After the electrochemical deposition/accumulation step, the adsorptive cathodic stripping voltammetric measurement was executed. (ii) The conventional ex-situ preparation of BiFE (i.e., using constant potential) was carried out in a separate 0.1 M acetate buffer modification solution (pH 4.5) containing 5 ppm Bi(III). After bismuth film deposition at a constant potential of −1.0 V for 150 s, the BiFE was rinsed with purified water and was ready to use. (iii) The nanostructured nsBiFE was prepared in a 0.1 M acetate buffer modification solution (pH 4.5) containing 5 mg L^−1^ Bi(III) using in-our-laboratory developed and optimized multi-pulse galvanostatic deposition protocol encompassing 50 consecutive cycles; each cycle included a current pulse of −100 μA for 5 s (pulse time) followed by a current pulse of +10 μA for 2 s (relaxation time). After modification, the nsBiFE was rinsed with purified water and was ready to use. The morphological differences of the ex-situ prepared BiFE and nsBiFE were presented in our recent publication [21].

Adsorptive cathodic stripping voltammetric (AdCSV) measurements of progesterone were carried out in a 0.1 M Na-PBS in the presence of dissolved oxygen. After electrochemical accumulation (at −1.0 or at −0.8 V), the stirring was stopped, and following equilibration step of 15 s, a stripping voltammogram was recorded using a square-wave potential scan in cathodic direction with a frequency of 25 Hz, a potential step of 8 mV, and an amplitude of 40 mV. 

## 3. Results and Discussion

**Figure 1:**
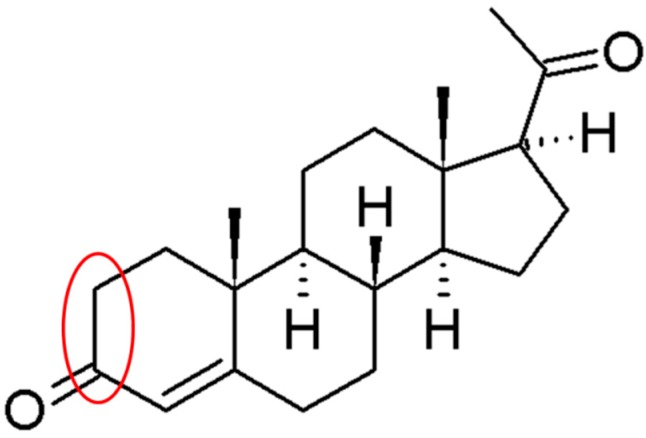
Chemical structure of progesterone and its electrochemically active sites marked in red.

Initial experiments involved the investigation of nsBiFE considering its capabilities of detecting low concentration levels of progesterone, as shown in Figure 1, together with its electrochemically active sites marked in red. This is in combination with adsorptive cathodic stripping voltammetry and the comparison with electroanalytical performances of an unmodified glassy carbon electrode (GCE), in situ prepared bismuth film electrode (BiFE) and ex situ prepared BiFE.

**Figure 2:**
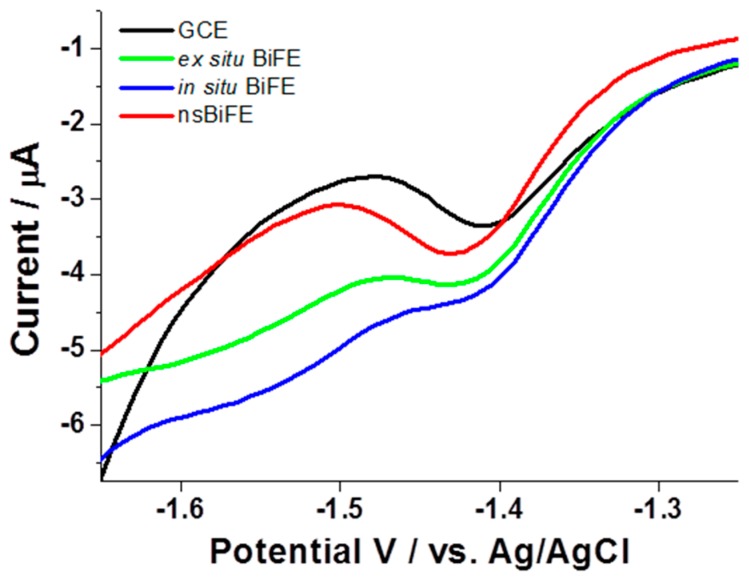
Adsorptive cathodic stripping voltammograms (AdCSVs) of 2.5 μmol L^−1^ progesterone recorded in 0.1 M Na-PBS at unmodified GCE (black), ex situ prepared bismuth film electrode (BiFE) (green), in situ prepared BiFE (blue) and at nanostructured BiFE (red) using accumulation potential of −1.0 V and accumulation time of 120 s.

The comparison was carried out in a 0.1 Na-PBS with pH adjusted to 7.1 aimed at approaching the pH of a real physiological sample, e.g., serum. As can be seen in Figure 2, all four investigated electrodes revealed stripping signals corresponding to 2.5 μmol L^−1^ progesterone at ca. −1.4 V with the highest signal obtained at the nsBiFE (red curve). The signal recorded with nsBiFE at −1.42 V was well-developed over the slightly sloped background where the beginning of hydrogen reduction reaction did not interfere with the analytical signal of progesterone. As expected, in the case of all three bismuth-based electrodes, the background was similar and not considerably affected by hydrogen reduction reaction, and the commencement of hydrogen reduction reaction appeared at less negative potentials when using unmodified GCE (black curve). In addition, the in situ prepared BiFE (blue curve) exhibited the poorest response among all compared electrodes due to the introduction of bismuth ions directly into the sample solution with a pH of 7.1, where the hydrolysis occurred, thus preventing optimal in situ formation of bismuth film. This effect can be partially mitigated with the addition of an auxiliary ligand, e.g., tartrate, into the measurements solution; however, this was not the focus of this preliminary study.

**Figure 3:**
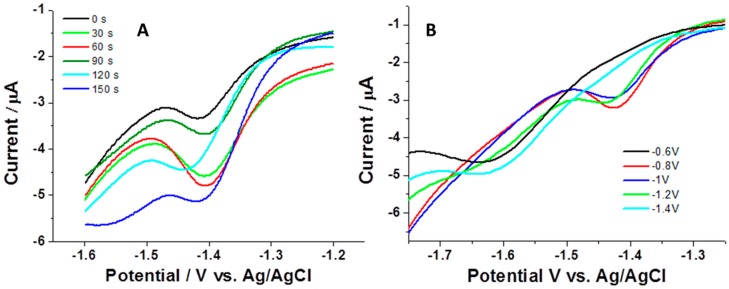
AdCSVs of 5.0 μmol L^−1^ progesterone recorded at nsBiFE using accumulation times of 0 s (black), 30 s (green), 60 s (red), 90 s (dark green), 120 s (light blue) and 150 s (blue) (**A**), and AdCSVs of 2.0 μmol L^−1^ progesterone recorded at nsBiFE using accumulation potentials of −0.6 V (black), −0.8 V (red), −1.0 V (blue), −1.2 V (green), and −1.4 V (light blue) (**B**). Other conditions are as in Figure 2.

The next examined parameter was the accumulation time. In this study, we followed the response of nsBiFE, i.e., current peak height, while changing the time of electrochemical accumulation. As demonstrated in Figure 3A, the signal of 5.0 μmol L^−1^ progesterone increased considerably when increasing the accumulation time from 0 to 60 s, and then attenuated at prolonged times up to the investigated 150 s accumulation. The attenuation of the signal (instead of leveling off) can be attributed to the saturation of the electrode surface and possible formation of multi layers, as also reported elsewhere [22], and is associated with lower conductivity. It should be stressed that lower concentrations of progesterone would allow longer accumulation times, i.e., later occurrence of saturation, and the saturation would occur even at shorter accumulation periods when using higher concentrations.

To obtain more insights into the electroanalytical behavior of nsBiFE, the effect of accumulation potential upon the signal of progesterone was monitored. As depicted in Figure 3B, the signal of progesterone (current peak height) increased with increasing the accumulation potential in a range of −0.6 to −1.2 V with the highest signal obtained at −0.8 V (red curve) and with practically no readable signal at −0.6 V. When the accumulation potential was more negative than −1.2 V, the signal decreased almost completely due to beginning of analyte reduction making the analyte accumulation insufficient; reduction of progesterone results in dimeric species [4] which tend to adsorb onto the electrode surface, thus attenuating its conductivity.

Based on these observations, the accumulation potential of −0.8 V was set as optimal, together with an accumulation time of 60 s to prevent the saturation of the analyte at the electrode surface and to attain the highest stripping voltammetric signal.

**Figure 4:**
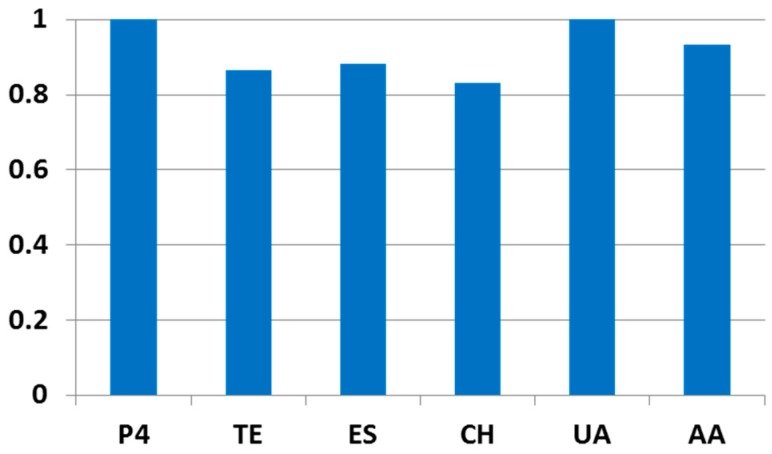
The effect of interfering species upon the signal of 0.5 μmol L^−1^ progesterone (P4); 50.0 μmol L^−1^ uric acid (UA), 50.0 μmol L^−1^ ascorbic acid (AA), 1.0 μmol L^−1^ testosterone (TE), 1.0 μmol L^−1^ 17-β-estradiol (ES) and 2.5 μmol L^−1^ cholesterol (CH).

In the next study, sensor performance was investigated in the presence of selected possible interfering compounds in concentration levels expected for women’s serum. For this purpose, the stripping voltammetric response of 0.5 μmol L^−1^ progesterone (P4) was monitored after an addition of 50.0 μmol L^−1^ uric acid (UA), 50.0 μmol L^−1^ ascorbic acid (AA), 1.0 μmol L^−1^ testosterone (TE), 1.0 μmol L^−1^ 17-β-estradiol (ES) and 2.5 μmol L^−1^ cholesterol (CH) into the measurement solution (Na-PBS). The change of signal heights for progesterone after addition of each interfering species is depicted in Figure 4; evidently, the addition of uric acid did not exhibit any readable effect upon the signal of progesterone, whereas the additions of ascorbic acid, testosterone, 17-β-estradiol, and cholesterol caused the signal attenuations of ca. 7%, 14%, 12%, and 17%, respectively. These results imply favorable selectivity of nsBiFE.

**Figure 5:**
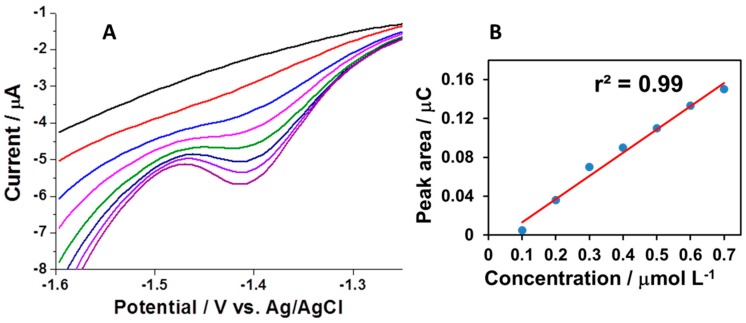
AdCSVs for successive additions of progesterone in 0.1 μmol L^−1^ steps together with background response obtained at nsBiFE using accumulation potential of −0.8 V for 60 s (**A**) and corresponding calibration plot (**B**). Other conditions are as in Figure 2.

Further characterization of the nanostructured bismuth film was conducted through investigating the response of nsBiFE while consecutively increasing the concentration of progesterone in a sub-micromolar range of 0.1–0.7 μmol L^−1^, which also corresponds to clinically significant levels of progesterone in serum during early pregnancy. The response is presented as a signal area corresponding to a passed charge, i.e., the integration of current over time. The bismuth-based sensor exhibited good linear behavior in the examined concentration range with a correlation coefficient r^2^ of 0.99 (inset), as shown in Figure 5.

Evidently, most of the electrochemical sensors for progesterone were studied in alkaline solutions using metal electrodes, such as mercury-, thin- and bismuth-based electrodes, and non-metal electrodes, such as imidazole-functionalized graphene oxide, some of them even in the presence of CTAB to facilitate progesterone reduction and its further dimerization. All of these sensors exhibited linear responses in similar or in higher concentrations ranges in comparison with the nsBiFE sensor. In addition, the nsBiFE sensor operates in a neutral medium. On the other hand, Moline et al. [20] reported on a sensitive electrochemical immunosensor for bovine serum progesterone; its preparation involves monoclonal antibody, cysteamine, gold nanoparticles, and measurements using horseradish peroxidase-labeled progesterone in the presence of pyrocatechol and hydrogen peroxide that makes preparation of the sensor as well as sensing procedure relatively complex. Herein, we present a simple preparation of progesterone sensor based on a nanostructured bismuth film electrode, which exhibits a linear response in concentration range significant for clinical diagnostics and operation under physiological pH conditions. The demonstrated performances can make the nsBiFE, after additional optimization and adaptation, potentially applicable as a sensor for measuring progesterone in pharmaceutical formulations and/or even as a rapid, disposable, point-of-care clinical diagnostic tool for monitoring progesterone during pregnancy.

## 4. Conclusions

In summary, we studied a nanostructured bismuth film electrode (nsBiFE) for convenient adsorptive cathodic stripping voltammetric detection of progesterone, which is an important hormone involved, among others, in the time of pregnancy assuring proper inhibition of uterus’ muscles activity. The nsBiFE revealed well-developed signal of progesterone and exhibited favorable electroanalytical performance, and most importantly, functioning in a medium with pH close to that of a real physiological environment. Such characteristics can provide a potential future applicability of this sensor as a simple, fast, and non-expensive portable monitoring/diagnostic tool.
